# Vacuum Casting and Mechanical Characterization of Nanocomposites from Epoxy and Oxidized Multi-Walled Carbon Nanotubes

**DOI:** 10.3390/molecules24030510

**Published:** 2019-01-31

**Authors:** Gerald Singer, Philipp Siedlaczek, Gerhard Sinn, Patrick H. Kirner, Reinhard Schuller, Roman Wan-Wendner, Helga C. Lichtenegger

**Affiliations:** 1 Institute of Physics and Materials Science, University of Natural Resources and Life Sciences Vienna, Peter-Jordan-Straße 82, 1190 Vienna, Austria; gerald.singer@boku.ac.at (G.S.); philipp.siedlaczek@boku.ac.at (P.S.); gerhard.sinn@boku.ac.at (G.S.); patrick.kirner@students.boku.ac.at (P.H.K.); reinhard.schuller@boku.ac.at (R.S.); 2 Department of Structural Engineering, Ghent University, 9000 Ghent, Belgium; roman.wanwendner@ugent.be

**Keywords:** vacuum casting, carbon nanotubes (CNTs), nanocomposite, mechanical properties

## Abstract

Sample preparation is an important step when testing the mechanical properties of materials. Especially, when carbon nanotubes (CNT) are added to epoxy resin, the increase in viscosity complicates the casting of testing specimens. We present a vacuum casting approach for different geometries in order to produce specimens from functional nanocomposites that consist of epoxy matrix and oxidized multi-walled carbon nanotubes (MWCNTs). The nanocomposites were characterized with various mechanical tests that showed improved fracture toughness, bending and tensile properties performance by addition of oxidized MWCNTs. Strengthening mechanisms were analyzed by SEM images of fracture surfaces and in-situ imaging by digital image correlation (DIC).

## 1. Introduction

Carbon nanotubes (CNTs) can be used as nanoscale fillers in order to improve the mechanical, electrical and thermal properties of polymers. The resulting so-called nanocomposites display significantly enhanced performance for many applications. For instance as electrically conductive and transparent flexible foils prepared from single-walled carbon nanotubes (SWCNTs) in poly(methyl methacrylate) (PMMA) [1], black paints with very high absorption of light [2] or as matrix components of carbon fiber reinforced composites (CFRPs) with increased stiffness and strength [3,4]. Also the dielectric properties and thermal conductivity of nanocomposites are of importance [5] as well as the electromagnetic wave absorption behavior of composites for high-temperature applications [6,7]. Nanocomposites are also of interest for fiber reinforced composites, thermosets and the particular CNT/epoxy materials. It is well known that surface-functionalization of CNTs and processing are highly important to benefit from the excellent properties of CNTs in a composite material [8]. Many different approaches have been developed in order to maximize the effect of reinforcement. For an epoxy matrix, the functionalization of CNTs with amines [9] or epoxy groups [10,11] is widely used, since good compatibility with the matrix can be achieved. Direct binding between reactive groups and the resin or interfacial adhesion is necessary to improve load-transfer within the nanocomposite, since the graphitic surface of CNT is generally inert and only provides weak interactions. It was shown that the effectiveness of the load-transfer mechanism between functionalized CNT and a polymer matrix can be quantified by the shift of the Raman G band [12]. For low filler content it was shown that oxidized CNTs show similar performance in mechanical tests of CNT/epoxy to amine-functionalized CNTs [13]. The advantage of oxidized CNTs is that oxidation can be carried out in a single step, whereas other functionalization treatments require additional reactions that make the process more complex. Usually, further cleaning steps are required in order to remove introduced contamination from the oxidizing agents.

In the present study, oxidized MWCNTs, produced by a “green” process [3], were dispersed in epoxy resin by calendering on a three-roll mill (TRM), which is one of the most successful techniques for the dispersion of CNTs in viscous matrices [14,15]. The resulting CNT/epoxy nanocomposites were mechanically tested in tensile and bending mode. Fracture mechanics of compact tension (CT) specimens were investigated and the fracture surface was studied by SEM.

Adding CNT to epoxies dramatically increases the viscosity and results in increased final porosity, which is an important aspect in the production of testing specimens. The formation of air bubbles during the casting procedure of specimens strongly influences the mechanical test results and has to be avoided. Therefore, in this study a vacuum casting technique was developed to produce high quality tensile specimens.

## 2. Results and Discussion

### 2.1. Dispersion

**Figure 1:**
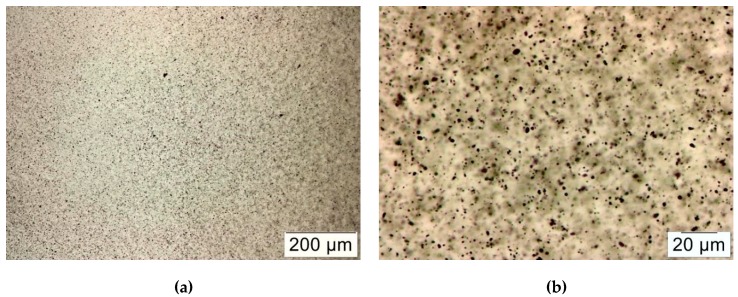
Optical light microscope images of oxidized MWCNT/epoxy dispersions at different magnifications, (**a**) 200 μm; (**b**) 20 μm.

The MWCNTs oxidized according to an oxidation procedure using 30% H_2_O_2_ aqueous solution [3], were dispersed in epoxy using a three-roll mill (TRM). The resulting dispersions of 0.5 wt% oxidized MWCNTs in epoxy resin were investigated under the optical light microscope. Figure 1a shows the homogenous distribution of oxidized MWCNTs in the epoxy matrix with a maximal agglomerate size of several microns in diameter (Figure 1b).

Calendering is a known technique for the production of dispersions from viscous resins and nanoparticles, e.g., epoxy with CNTs [8]. It was reported that three-roll milling leads to the existence of both individualized CNTs as well as agglomerates [15]. General information about the dispersion quality can be obtained from light microscopy images (Figure 1) and is typically judged by the size of remaining agglomerates (in this case <5 μm and thus judged as suitable for composite production).

### 2.2. Vacuum Casting Technique

**Figure 2:**
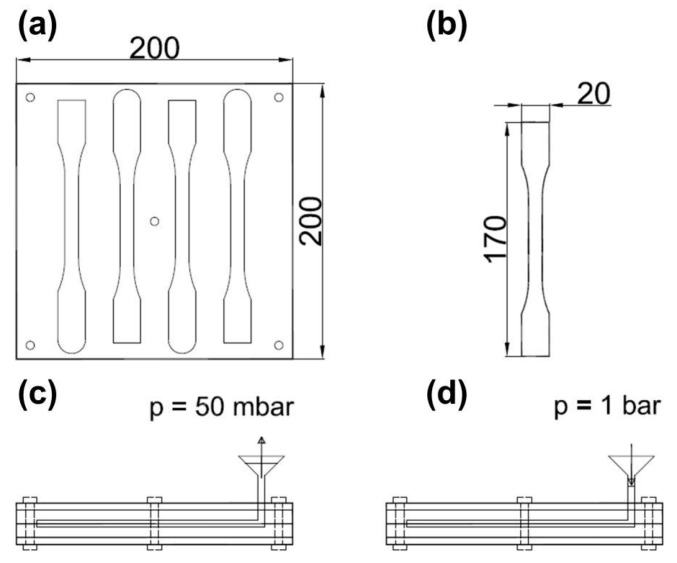
Principle of the vacuum casting technique for tensile specimens: (**a**) Silicone mold for four specimens and (**b**) their dimensions. (**c**) Evacuation of the closed mold with a filled funnel and (**d**) the flow of material inside the mold after ventilation of the vacuum chamber. All measures are given in mm.

Tensile specimens were cast in a closed silicone mold according to the illustration in Figure 2. First, funnels were attached to the mold and filled with ~15 g of the mixed components (resin + hardener) each. Then the mold was placed in a vacuum oven and evacuated to ~30 mbar. After the air was fully removed, the oven was carefully ventilated. Due to the atmospheric pressure, the material flows inside the silicone mold. The specimens were cured for 24 h at room temperature and were removed from the mold afterwards.

Tensile test specimens were much larger compared to the ones used for bending and CT tests and therefore the probability of bubbles within the relevant test area is higher. Furthermore, under tension the whole specimen is under load and will fail at the weakest part, which is given by porosity, whereas for bending and CT tests, only a certain area experiences the maximum load during testing.

### 2.3. Mechanical Testing

After the resin was mixed with the hardener component, specimens were casted for three different mechanical tests. Tensile specimens were cast using the vacuum casting technique as described in the Materials and Methods section and specimens for bending and CT tests were cast in open silicone molds. The CT specimens required additional drilling and cutting of the notch.

#### 2.3.1. Tensile Tests

Table 1 shows the results of tensile tests of CNT/epoxy nanocomposites (EP/CNT), containing 0.5 wt% oxidized MWCNTs, and neat epoxy (EP) as reference. Adding 0.5 wt% oxidized MWCNTs to the epoxy resin led to an increase of the Young’s modulus (*E*) by +8%, while the tensile strength (***σ***) was improved by +44%. Also the elongation at break (***ε***) increased by almost +33% compared to neat epoxy. A small reduction of the Poisson’s ratio (*ν*) by +6% was obtained for CNT/epoxy nanocomposites, which means less contraction in the width of the specimen under axial tensile load. The elastic parameters *E* and *µ* were evaluated by digital image correlation (DIC) measurements.

Improvement of the Young’s modulus by +8% was obtained in this study, which is in the lower range to what was reported in literature, whereas the increases in tensile strength and elongation at break are relatively high [16]. In general, the tensile properties of the CNT/epoxy nanocomposite strongly depend on the amount of filler and their functionalization, the dispersion technique as well as the type of CNT that is used. Particle loadings between 0.1 wt% up to several wt% have been proven to effectively reinforce an epoxy matrix [16]. In a different study relatively high loading of 6 wt% MWCNT were added to epoxy in order to compare the effect on tensile properties of the resulting nanocomposites [17]. The Young’s modulus was improved from 3.1 GPa (unreinforced epoxy) to 3.6 GPa and 4.1 GPa by pristine and oxidized MWCNTs, respectively. However, the fracture strength and strain was reduced compared to the reference. At lower CNT loading comparable tensile strength to pure epoxy was reported, while the Young’s modulus was improved by +10% (0.1% MWCNTs) and +18% (0.2% MWCNTs).

**Figure 3:**
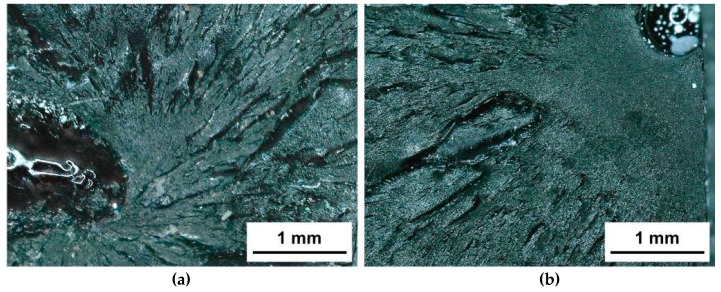
Optical light microscope images of the fracture surface of porous tensile specimens: Initiation of the crack due to an air bubble (**a**) in the inside of a specimen and (**b**) at the surface of the specimen.

As a comparison to properly prepared tensile specimens using the presented vacuum technique, mechanical tests of porous specimens that were cast in open silicone molds were performed. The results of specimens with pores were significantly lower and the scattering of the mean values of four tested specimens for Young’s modulus and tensile strength was higher (*E* = 3420 ± 415 MPa. *σ* = 27.2 ± 10.3 MPa, *ν* = 0.38 ± 0.03, *ε* = 0.97 ± 0.32%) compared to regular specimens. It has to be mentioned that such porous specimens usually have to be excluded from mechanical tests and in this case the samples are used for comparison only. Figure 3 shows the fracture surface of porous tensile specimens. It can be observed that porosity that may exist in the inside of the material (Figure 3a) or at the surface (Figure 3b) initiates the failure.

#### 2.3.2. Four-point Bending

The flexural modulus (*E_f_*) was evaluated by DIC measurements and shows an improvement of +12% by adding oxidized MWCNTs to the resin (Table 2). Much higher flexural strength (*σ_f_*) and elongation at break (*ε_f_*) was obtained for nanocomposites with increases of +49% and +84%, respectively.

Similar to the tensile properties, also in bending mode the flexural modulus was improved significantly less compared to the flexural strength and elongation at break. This behavior suggests that the reinforcement mechanism acts more effectively in the plastic deformation than in the elastic range.

#### 2.3.3. Compact Tension (CT) Tests

**Figure 4:**
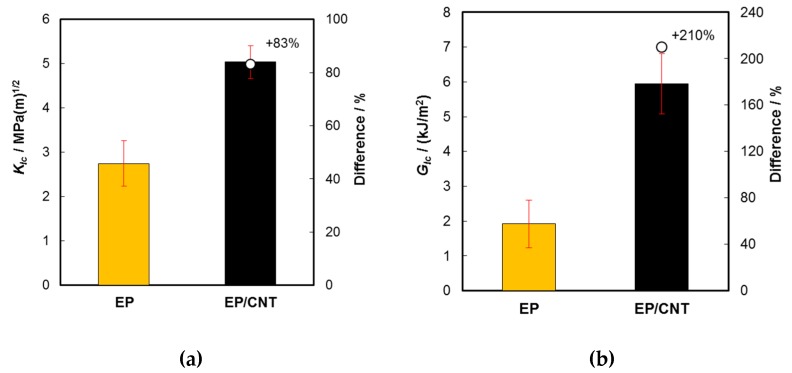
Results obtained from CT tests of neat epoxy (EP) and CNT/epoxy nanocomposites (EP/CNT): (**a**) fracture toughness (*K_Ic_*) and (**b**) critical strain energy release rate (*G_Ic_*).

Results of CT tests are shown in Figure 4. The fracture toughness (*K_Ic_*) of investigated nanocomposites was increased by +83% (Figure 4a) and the critical strain energy release rate (*G_Ic_*) was improved by +210% (Figure 4b). *G_Ic_* was calculated using the following equation:

(1) $$G_{Ic}=\frac{\left(1-\nu^{2}\right)K_{Ic}^{2}}{E}$$

Compared to other epoxy resins, the fracture toughness and the critical strain energy release rate of the unmodified and the CNT-modified epoxy in the present study were relatively high. However, the extent of improvement is not unusual when a proper surface functionalization of the CNT is used [16]. Crack bridging, pull-out of CNT and inner tubes as well as rupture of CNT were reported as the main toughening mechanisms in MWCNT/epoxy nanocomposites [18].

### 2.4. SEM Investigations

**Figure 5:**
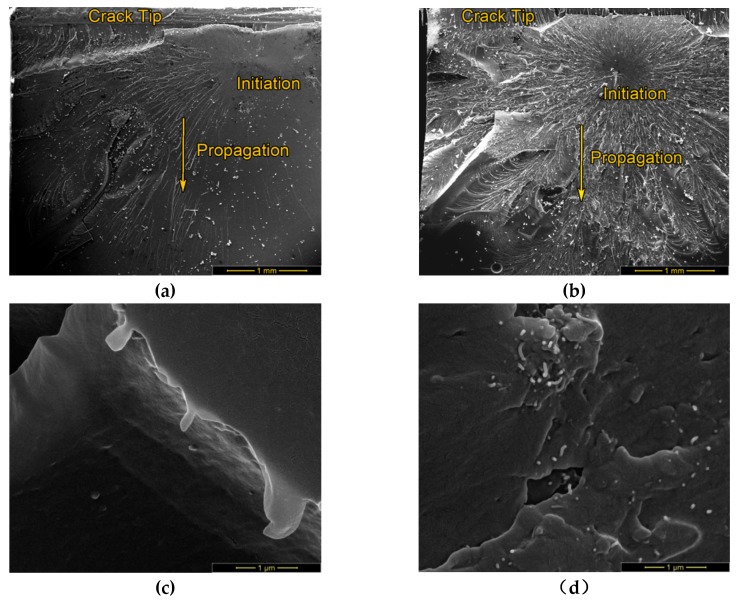
Micrographs from neat epoxy and CNT/epoxy. (**a**) Neat epoxy: Crack initiation started at the edge of the crack tip. (**b**) CNT/epoxy: Crack initiation started 0,5 mm away from the edge. (**c**) Neat epoxy: higher magnification reveals smooth surface. (**d**) CNT/epoxy: higher magnification shows plastic deformation at carbon nanotube agglomerates.

The micrographs in Figure 5 show fracture surfaces of CT specimens of neat epoxy and CNT/epoxy composite at low (a, b) and high magnification (c, d). At low magnification the crack tip is located at the top of the image. The crack initiation point and propagation direction can be detected by microrivers, radiating from a center. In Figure 5b (CNT/epoxy composite) the crack initiated approximately 500 μm from the crack tip, which indicates a strong bonding at the predetermined breaking point. This appearance is not visible in the sample without CNT (Figure 5a), where the crack initiated directly at the crack tip.

The most obvious difference between neat epoxy and CNT/epoxy composite is the surface roughness around the crack initiation point. It seems that CNT-filled epoxy composite (Figure 5b) had undergone high plastic deformation, which is usually not common for brittle thermoset polymers. From [15] it is known that there are two dominant mechanisms that describe fracture deformation behavior in polymers, which are shear yielding and crazing. Shear yielding is defined by a molecular shear motion at constant volume which leads to plastic deformation, whereas crazing are microcracks originating from microvoids or inhomogeneities. While shear yielding is significant in CNT-dispersed epoxy, sparse features are observed in neat epoxy, indicating a brittle fracture mechanism. Crazing is usually found in brittle thermoplastics and limits plastic deformation. However, multiple crazing leads to toughening and even ductile like behavior can be clearly observed in the CNT/epoxy composite (Figure 5b) as microcracks perpendicular to the propagation direction. Crazes could have arisen from CNT agglomerates, acting as inhomogenities that act as initiators for fine cracks.

At higher magnification (Figure 5d) modified CNT bundles are identified as well dispersed individual units or agglomerated bundles. Compared to neat epoxy (Figure 5c), high roughness and increased plastic deformation can be observed. According to Cha et al. [11], CNT can bridge cracks and increase the fracture toughness by CNT pulling-out. Those mechanisms can be utilized best, if CNT are oriented perpendicular to the crack growth, however randomly dispersed CNT could have a positive effect on fracture toughness already [16]. Rupture and pull-out effects can be exploited in a particularly effective way, if a strong interface between matrix and CNT is provided, e.g., by oxidation of CNT. Since almost all CNTs, protruding out of the resin, appear to have similar length, it can be suggested that CNT pull-out and subsequent rupture was the main mechanism to absorb fracture energy.

The described failure mechanisms were observed for all specimens, suggesting the typical effect of CNT-reinforcement in this case is based on effective load-transfer between oxidized MWCNT and epoxy matrix that results in increased fracture energy due to: (I) high load-bearing capacity of MWCNT, (II) energy-consuming pull-out of CNT and (III) crack branching, leading to higher roughness of the fracture surface and additional plastic deformation.

## 3. Materials and Methods

### 3.1. Nanocomposites

The epoxy resin was based on bisphenol-A/F epichlorohydrin with *m*-Phenylenebis- (methylamine) hardener. The mixing weight ratio of resin and hardener was 3:1. Pristine MWCNTs (NC7000, Nanocyl, Sambreville, Belgium) with average diameter of ~9.5 nm and 1.5 µm length were oxidized using 30% H_2_O_2_ aqueous solution according to a previously developed procedure [3]. A pre-dispersion of 0.5 wt% oxidized MWCNT in epoxy resin was produced with a mechanical stirrer. In the next step the fine dispersion was done on a three-roll mill (type 80E, Exakt, Norderstedt, Germany) using the parameters given in Table 3.

### 3.2. Mechanical Testing

For tensile tests the vacuum cast specimens according to DIN EN ISO 527 were ground to 4 mm and sprayed with a speckle pattern using white undercoating and black dot pattern. A spindle-driven testing frame (Zwick/Roell, 10 kN, Ulm, Germany) was used at a constant testing speed of 5 mm/min. Four tensile specimens were tested of each type. The surface deformation of the specimen was recorded with a digital image correlation (DIC) system (Q-400, Dantec Dynamics, Skovlunde, Denmark), consisting of two cameras. The images were processed using Istra 4D V4.4.4.694 to calculate the surface mean principal strains.

Specimens for four-point bending tests were casted in an open silicone mold. The dimensions of the bending specimens were 80 × 15 (mm²) and the thickness was ground to 4 mm. The distance of the support span of the fixture was 66 mm and 22 mm for the loading span. A least four specimens were tested of both, neat and CNT-reinforced epoxy. As already described for the tensile specimens a speckle pattern was created on the side of the specimen in order to record the deformation with DIC. Loading speed was set constant to 2 mm/min.

**Figure 6:**
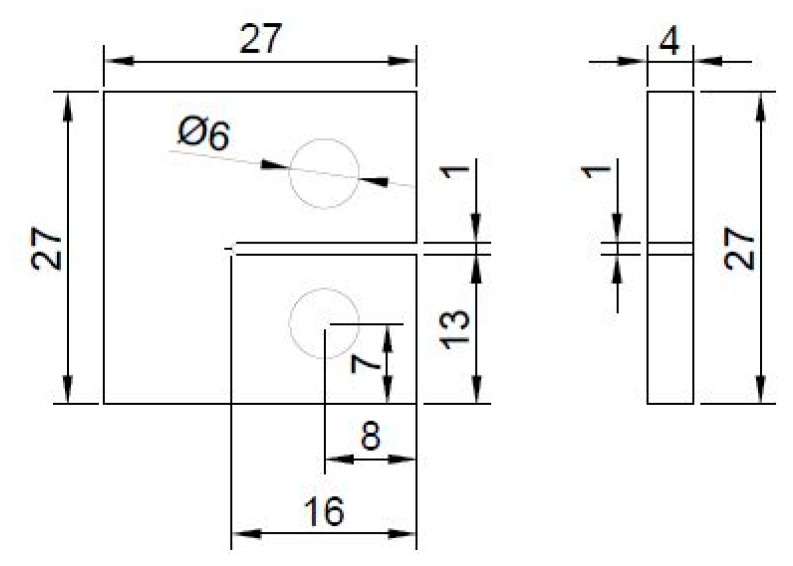
Dimensions (in mm) of the compact tension (CT) specimen.

The compact tension (CT) specimens were casted in an open silicone mold, ground to 4 mm thickness, drilled and notched according to the dimensions in Figure 6. In order to produce precise holes, an in-house built metal template was used in which the specimen was fixed. The notch was cut on a circular saw and before testing, a fine crack was initiated with a fresh blade. The strain was measured with a clip-gage that was attached directly to the specimen on the side of the notch. Force was applied with a constant speed of 0.5 N/mm. For both testing series at least five CT specimens were tested.

### 3.3. Scanning Electron Microscopy (SEM)

For the SEM investigations a 250 FEG (FEI Quanta, Thermo Fisher Scientific, Hillsboro, OR, USA) was used under high vacuum and secondary electron (SE) mode. The fracture surface was sputtered with a thin layer of gold in order to provide sufficient electrical conductivity. Accelerating voltages between 20–30 kV were used for imaging.

## 4. Conclusions

In this study, a vacuum casting technique for tensile specimens of MWNCT/epoxy nanocomposites was presented as a novel sample preparation method, which is a critical step and usually very challenging due to the high viscosity of the resin. Results of tensile tests were compared to a regular casting technique where porous specimens and reduced properties were observed, which highlights the importance of proper sample preparation. It was shown that adding oxidized MWCNTs to epoxy resin may significantly improve the mechanical properties of the resulting MWCNT/epoxy nanocomposite. In this case, the elastic properties were slightly improved, while the strength and elongation at break were increased in a much more pronounced way. Obviously, the reinforcing mechanism is more important in the plastic deformation than in the elastic region. The fracture toughness of MWCNT-reinforced epoxy and the critical strain energy release rate were enhanced substantially. The fracture surface of CT specimens was investigated with SEM, revealing higher plastic deformations and a rougher surface morphology of MWCNT/epoxy nanocomposite compared to neat epoxy. Furthermore, probably due to the suitable surface modification by oxidation of the CNT, a substantial amount of crack bridging and fiber pull-out was observed, resulting in increased fracture energy and thus improved strength and fracture toughness of MWCNT/epoxy nanocomposites.

## Figures and Tables

**Table 1:** Results from tensile tests of neat epoxy (EP) and CNT/epoxy nanocomposites, containing 0.5 wt% oxidized MWCNT (EP/CNT). Mean values are given together with the standard deviation (SD).

	*E* (MPa)	SD	*ν* (-)	SD	*σ* (MPa)	SD	*ε* (%)	SD
**EP**	3366	153	0.41	0.02	30.2	2.3	1.12	0.12
**EP/CNT**	3647	150	0.39	0.05	43.5	4.6	1.49	0.21

**Table 2:** Four-point-bending test results of neat epoxy resin and CNT/epoxy nanocomposites. The numbers represent mean values and the standard deviation (SD).

	*E_f_* (MPa)	SD	*σ_f_* (Mpa)	SD	*ε_f_* (%)	SD
**EP**	3045	153	66.2	15.5	1.99	0.41
**EP/CNT**	3401	144	98.5	10.2	3.66	0.89

**Table 3:** Dispersion parameters on the three-roll mill.

Step	Gap 1	Gap 2
1	120 µm	40 µm
2	30 µm	10 µm
3	15 µm	5 µm
4	5 µm	10 N/mm
